# New frontiers of oral sciences: Focus on the source and biomedical application of extracellular vesicles

**DOI:** 10.3389/fbioe.2022.1023700

**Published:** 2022-10-19

**Authors:** Wenting Yu, Shengnan Li, Guohao Zhang, Hockin H. K. Xu, Ke Zhang, Yuxing Bai

**Affiliations:** ^1^ Department of Orthodontics, Beijing Stomatological Hospital, School of Stomatology, Capital Medical University, Beijing, China; ^2^ Department of Oral and Maxillofacial Surgery, Peking University School and Hospital of Stomatology and National Center of Stomatology and National Clinical Research Center for Oral Diseases and National Engineering Research Center of Oral Biomaterials and Digital Medical Devices, Beijing, China; ^3^ Biomaterials and Tissue Engineering Division, Department of Advanced Oral Sciences and Therapeutics, University of Maryland School of Dentistry, Baltimore, MD, United States; ^4^ Center for Stem Cell Biology and Regenerative Medicine, University of Maryland School of Medicine, Baltimore, MD, United States; ^5^ Marlene and Stewart Greenebaum Cancer Center, University of Maryland School of Medicine, Baltimore, MD, United States

**Keywords:** extracellular vesicles, exosomes, oral cavity, source, EVs-based biomedical application

## Abstract

Extracellular vesicles (EVs) are a class of nanoparticles that are derived from almost any type of cell in the organism tested thus far and are present in all body fluids. With the capacity to transfer “functional cargo and biological information” to regulate local and distant intercellular communication, EVs have developed into an attractive focus of research for various physiological and pathological conditions. The oral cavity is a special organ of the human body. It includes multiple types of tissue, and it is also the beginning of the digestive tract. Moreover, the oral cavity harbors thousands of bacteria. The importance and particularity of oral function indicate that EVs derived from oral cavity are quite complex but promising for further research. This review will discuss the extensive source of EVs in the oral cavity, including both cell sources and cell-independent sources. Besides, accumulating evidence supports extensive biomedical applications of extracellular vesicles in oral tissue regeneration and development, diagnosis and treatment of head and neck tumors, diagnosis and therapy of systemic disease, drug delivery, and horizontal gene transfer (HGT). The immune cell source, odontoblasts and ameloblasts sources, diet source and the application of EVs in tooth development and HGT were reviewed for the first time. In conclusion, we concentrate on the extensive source and potential applications offered by these nanovesicles in oral science.

## 1 Introduction

**FIGURE 1:**
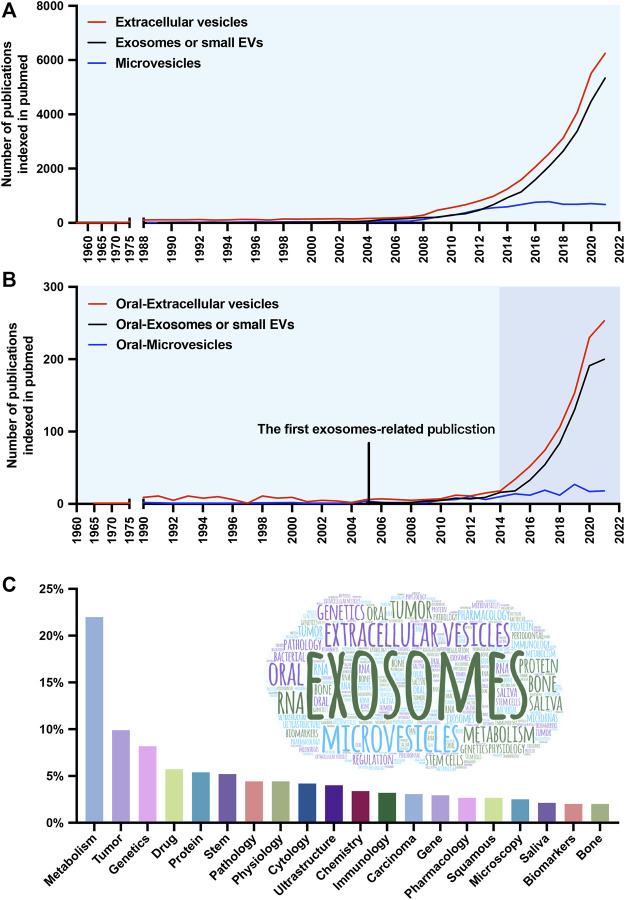
Number and keyword analysis of publications. **(A)** Timeline (1960–2021) of the publications referring to extracellular vesicles (EVs) (red line), exosomes or small EVs (black line), and microvesicles (blue line). **(B)** Timeline of the publications referring to oral-extracellular vesicles (red line), oral-exosomes or small EVs (black line), and oral-microvesicles (blue line). **(C)** The frequency distribution and cloud maps of keywords in the publications.

Extracellular vesicles (EVs) research, particularly exosomes research, is one of the most rapidly growing biomedical fields. This area of research has attracted extensive attention recently due to EVs having the capacity to transfer “functional cargo and biological information” to regulate local and distant intercellular communication and the potential of EVs as diagnostic and therapeutic tools for the treatment of diverse diseases and the adjustable of EVs functions. The term “EVs” encompasses a heterogeneous group of cell-derived membrane vesicles that are present in all body fluids due to membrane shedding by any cell type in the organism tested thus far, including bacteria (van der Pol, et al., 2012). The first story about these tiny phospholipid bilayer-covered particles can be traced back to 1946, with EVs were referred to pro-coagulant platelet-derived particles (Chargaff and West, 1946). Later, Peter Wolf called EVs “platelet dust”, referring to them as a garbage bin (Wolf, 1967). Since the 1970s–1980s, EVs biology began to attract more attention. Researchers claim that EVs can be found in serum (Benz and Moses, 1974) and the cell medium of reticulocytes (Johnstone, et al., 1987). Promisingly, scientists in the field of oral science isolated extracellular vesicles from *bacteroides gingivalis* at almost the same time (Grenier and Mayrand, 1987). Although related studies started early, they were interrupted, and at least another 15 years elapsed until investigators refocused on the oral region, primarily exosomes in saliva (Kapsogeorgou, et al., 2005) (Figure 1). The oral cavity is a special part of the human organ. It includes multiple types of tissue, is the beginning of the digestive tract, and it is also harboring up to 1,000 bacterial species that maintenance of both oral and systemic health (Soro, et al., 2014). The research on EVs in oral cavity is important because complexity and particularity of oral function. However, a literature search revealed few systematic review on the source and biomedical applications of EVs in oral cavity.

According to their size distribution and biogenesis, EVs can be divided into exosomes, microvesicles (MVs), and apoptotic bodies. Exosomes are the most specialized subgroup, generated by the inward budding of endosomal membranes and having a minimum diameter (almost 30–150 nm) in EVs. In contrast, MVs (100 nm-1 μm) and apoptotic bodies (1–5 μm) originate from outward budding (Akers, et al., 2013; Tkach and Thery, 2016). Notably, these sizes overlap, so it is challenging to separate these three subgroups both in experiments and descriptions. Moreover, the protein markers used to identify exosomes (such as ALIX and TSG101) can be found in MVs. Therefore, the International Society for Extracellular Vesicles suggested EVs as a preferential term used to describe all the previously mentioned types (Gould and Raposo, 2013), and the term “small EVs” is sometimes used to describe the exosomes. This review mainly concentrates on exosomes and microvesicles, and EVs serve as a general term referring to all subtypes.

Growing evidence suggests that extracellular vesicles exert their function mainly *via* the targeted transfer of functional cargo to promote intercellular communication. Novel biological functions of EVs continue to be described, including cancer treatment, early diagnosis, tissue regeneration, and drug delivery (Huang, et al., 2019; Wiklander, et al., 2019; Kalluri and LeBleu, 2020). Research also shows that mesenchymal stem cell (MSC)-derived exosomes have no adverse effects on toxicological testing (Ha, et al., 2020a). Even xenogeneic extracullular vesicles have shown a therapeutic effect similar to that of allogeneic EVs in soft tissue repair (Dong, et al., 2020). These findings imply that the extracellular vesicles not only exhibit potential for clinical applications but are also relatively safe. Therefore, the objectives of this paper were to review current knowledge on both cell source and cell-independent source of EVs in oral cavity, highlight the research directions of biomedical application of EVs, and elucidate the mechanisms of extracellular vesicles-based horizontal gene transfer for the first time.

## 2 Isolation and characterization of extracellular vesicles

**FIGURE 2:**
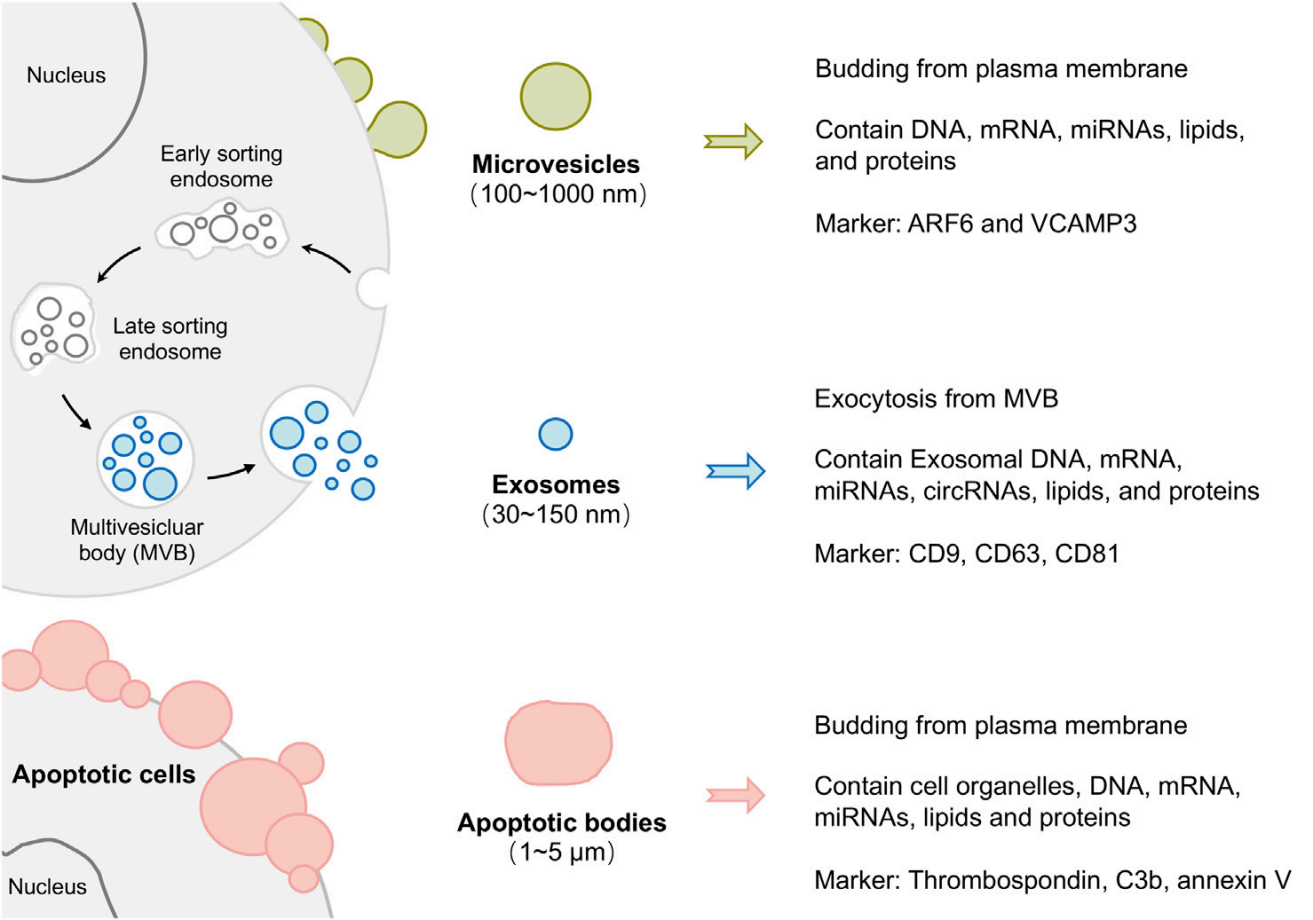
Schematic representation of extracellular vesicle (EVs) biogenesis and heterogeneity. According to their size distribution and biogenesis, EVs can be divided into exosomes, microvesicles, and apoptotic bodies.

Although exosomes, microvesicles, and apoptotic bodies are all membrane trafficking vesicles, they have a totally different biogenesis pattern. As shown in Figure 2, Exosomes originate through endocytosis, which means that the plasma membrane invaginates from outside to inside to raise vesicles. As a result, exosomes contain extracellular materials and cellular membrane constituents (Hessvik and Llorente, 2018). MVs and apoptotic bodies, conversely, are generated *via* outward budding by pinching off from the plasma membrane surface (inside to outside). Therefore, MVs and apoptotic bodies are known collectively as ectosomes (Colombo, et al., 2014; Teng and Fussenegger, 2020).

It is usually considered that EVs biofunctions based on their specific bioactive cargo, including lipids, proteins and genetic material. The lipids usually have the same function, providing structural stability, encapsulating EVs functional cargo and protecting it from enzymatic digestion (Kourembanas, 2015). The proteins in EVs reflect the vesicle’s mechanism of biogenesis and fusion, and also serve as markers to identify EVs and represent their cellular origin (Simpson, et al., 2008; Keerthikumar, et al., 2016). The functional genetic materials, including RNA (mRNA, miRNA, and other noncoding RNA), DNA, and other cytosolic molecules and ingredients. Several studies have shown that packed genetic material in EVs is exchanged between cells and subsequently translated to induce the reprogramming of EVs target cells (Valadi, et al., 2007; Ekstrom, et al., 2012). Numerous researchers have applied themselves to providing a more exhaustive and comprehensive characterization of EVs content. There are at least two public online databases: Evpedia (Kim, et al., 2013) and Vesiclepedia (Pathan, et al., 2019) (previously Exocarta), which are constantly updated and are crucial tools to improving our understanding of the EVs complexity.

It is worth noting that disease-related and specific active genetic molecules can be encapsulated in EVs. Hence, there are two promising directions for the application of EVs. On the one hand, in light of the RNA content of EVs changes with the pathological condition, they have become an interesting origin of biomarkers for diagnosing human disease (Momen-Heravi et al., 2018). On the other hand, exploiting the biological characteristics that EVs are able to modulate the phenotype and behavior of recipient cells, EVs are widely applied in disease therapeutics (Phinney and Pittenger, 2017; Lasser, et al., 2018).

## 3 Sources of extracellular vesicles in the oral cavity

The biological efficacy of extracellular vesicles mainly depends on the cellular origin and physiological condition of the parent cells (Tkach and Thery, 2016). Thus, the potential sources of EVs in the oral cavity and the specificity of their donor cells are discussed in detail in this section. To appreciate the EVs in oral cavity, the complexities and particularities of oral cavity need to be taken into consideration first. The mouth cavity itself is complex, including various hard tissue (teeth and bones), soft tissue (lip, cheek, tongue, and palate), nerves, and blood vessels (Madani, et al., 2014). Furthermore, the mouth is the beginning of the digestive tract through which numerous diets obtain access to the human body. It cannot be ignored that the oral cavity is one of the four major bacterial banks of the human body too (Gao, et al., 2018). There are two disparate sources may contribute to secrete EVs in oral cavity: cell source (dental tissue-derived cells, bone marrow mesenchymal stem cells, cancer cells, and immune cells) (Table 1) and cell-independent sources (microbiome, saliva, and diet) (Table 2)*.* In the following sections, each of these sources will be reviewed.

**TABLE 1:** The cell sources and features of extracellular vesicles (EVs).

Original of EVs	Recipient of EVs	Content profile	Functions	References
**Dental tissue-derived cells**				
GMSCs	Macrophages	CD73, miR-1260b	Modulation of the inflammatory phenotypes	Jiang and Xu (2020); Nakao, et al. (2020); Shi, et al. (2017); Wang, et al. (2020a)
	MC3T3-E1	TGF-β, VEGF	Bone regeneration	
			Wound healing	
PDLSCs	BMSCs	miRNAs	Bone regeneration	Kanjanamekanant, et al. (2014); Liu, et al. (2020a); Rajan, et al. (2016); Zhang, et al. (2020a)
	Macrophages	IL-10, TGF-β	Anti-inflammation and immunosuppressive effects	
	HUVECs	VEGFA	Angiogenesis	
	PDLs	IL-1b	Response mechanical stress	
DPCs	DPCs		Dental tissue regeneration	Couble, et al. (2000); Huang, et al. (2016); Wen, et al. (2021); Zhou, et al. (2020)
	ECs		Angiogenesis	
Odontoblasts and ameloblasts	Mineralized dentin	DPP	Construct well-mineralized tooth structures	Kidd and Fejerskov (2004); Matsuo, et al. (1986); Rabie and Veis (1995); Wang, et al. (2019a); Zhang, et al. (2014)
	ECM			
	SCAPs		Maintain tooth homeostasis	
SHEDs	BMSCs	miR-100-5p	Dental tissue regeneration	Jarmalaviciute, et al. (2015); Luo, et al. (2019); Zhuang, et al. (2020)
	Neural stem cells		Treatment Parkinson’s disease	
	Chondrocytes		Suppress inflammation in TMJ	
Other Cells	Such as Hertwig’s epithelial root sheath cells (HERS), periapical cyst-mesenchymal stem cells (PCy-MSCs), dental follicle cells (DFCs), oral keratinocyte (OKEx), oral mucosa lamina propria-progenitor cells (OMLP-PCs), and salivary gland epithelial cells. However, EVs from these sources have not been broadly studied due to the particularity of the cells.			Kapsogeorgou, et al. (2005); Shi, et al. (2020); Sjoqvist, et al. (2019a); Tatullo, et al. (2019); Yang, et al. (2020a); Zhang, et al. (2020b)
**BMSCs**	HUVECs	miR-146a	Bone regeneration	Davis, et al. (2017); Liu, et al. (2020b); Liu, et al. (2020c); Liu, et al. (2020d); Liu, et al. (2021); Yang, et al. (2020b)
	Macrophages		Wound healing	
	ECs	miR-125a, miR-125b	Improve cardiac function	
	PDLSCs		Immunomodulation	
	T cells	miR-183 cluster	Induces senescence	
	BMSCs			
**Cancer cells**				
OSCC	Stromal cells around cancer tissues	hsp90	Tumor treatment	Ono, et al. (2018); Zhao, et al. (2020)
ACC	HPLF	MRPL23-AS1	Facilitate tumor metastasis	Chen, et al. (2020); Xu, et al. (2019)
	Microvascular endothelial cells			
**Immune cells**				
Macrophages	BMSCs	miR-378a	Bone regeneration	Kang, et al. (2020); Liu, et al. (2020e)
T cells	Jurkat cells	microRNA-21	Immunomodulation	Dou, et al. (2020); Yang, et al. (2020b)
	Macrophages	Curcumin		

**TABLE 2:** The cell-independent sources and features of extracellular vesicles (EVs).

Original of EVs	Recipient of EVs	Content profile	Functions	References
**Microbiome**				
*P.g.*	HPLF	LPS	Accelerate ECM degradation prevents osteogenic differentiating	Fleetwood, et al. (2017); He, et al. (2020); Sang, et al. (2014); Singhrao and Olsen (2018)
	Macrophages		Active inflammation	
	Brain		Contribute to AD development	
	Lung epithelial cells		Induce cell death	
*A.a.*	HGF	CDT	Deliver virulence factors	Ha, et al. (2020a); Nice, et al. (2018); Rompikuntal, et al. (2012)
	THP-1 cells	LtxA	Cause neuroinflammation	
	Brain monocyte and microglial cells	IL-6 and TNF-α		
*G. adiacens*	PBMCs	Virulent proteins	Elicit inflammation	Alkandari, et al. (2020)
*S. mutans*	C. albicans		Facilitate bacteria cross-kingdom interactions	Wu, et al. (2020); Wu, et al. (2022b)
			Enhancing candida albicans cariogenic ability	
**Saliva**		DPP IV miRNA/proteins	Influence immune response	Chaparro Padilla, et al. (2020); Kim, et al. (2017); Li, et al. (2020); Yakob, et al. (2014)
			Diagnosis	
**Diet**				
milk	Osteocytes	CD9, CD81, NT5E, CD59	Bone regeneration	Oliveira, et al. (2020); Sadri, et al. (2020); Tong, et al. (2020)
	Murine placenta and embryos		Facilitate embryo survival	
	Gut Microbiota		Immune regulation	
Plant	P.g.	Lipids, PA, miR159a	Inhibit pathogenicity of P.g.	Sundaram, et al. (2019)
**Other sources**	EVs have also been isolated from gingival crevicular fluid, junctional epithelium, and periosteum. Perhaps because these sources are not representative, related researches are very limited.			Atsawasuwan, et al. (2018); Shimono, et al. (1991); Sun, et al. (2019)

### 3.1 Extracellular vesicles released by cell sources

#### 3.1.1 Dental tissue-derived cells

##### 3.1.1.1 Gingival mesenchymal stem cells

Gingival mesenchymal stem cells (GMSCs) can be isolated from gingival lamina propria. GMSCs are characterized by markedly reduced inflammation, notable fast wound-healing aptitude, and easily accessible during dental surgery (El-Sayed, et al., 2015). EVs in oral cavity were first discovered in GMSCs, and they can be traced back to 1990 (Trabandt, et al., 1990). GMSC-derived exosomes have been verified to induce anti-inflammatory M2 macrophage polarization and this effect can be reinforced through tumor necrosis factor-alpha (TNF-α) in the microenvironment (Wang, et al., 2020a; Nakao, et al., 2020). EVs derived from GMSCs also have great potential in tissue regeneration. GMSC-exosomes have been demonstrated the encapsulate of several growth factors, such as transforming growth factor-beta (TGF-β) and vascular endothelial growth factors (VEGF), promoting the migration and osteogenic differentiation of preosteoblast MC3T3-E1, and accelerating wound healing in the diabetic skin defect model (Shi, et al., 2017; Jiang and Xu, 2020).

##### 3.1.1.2 Periodontal ligament stem cells

Periodontal ligament stem cells (PDLSCs) are the most extensively studied EVs source in oral cavity. PDLSCs, a subgroup of cells from periodontal ligament, are considered a traditional source of multipotential stem cells to direct regeneration (Seo, et al., 2004). Derivatives of PDLSCs (such as culture mediums or EVs) have been confirmed to promote calvarial bone regeneration (Liu, et al., 2020a) and to possess immunomodulatory and neuroprotective effects in relapsing remitting multiple sclerosis (RR-MS) patients (Rajan, et al., 2016). Similarly, the inflammatory microenvironment has been shown to increase exosomes secretion and enhance VEGFA transfer in exosomes to promote angiogenesis in periodontal ligaments (Zhang, et al., 2020a). Moreover, PDLSCs are the critical response cells to mechanical force during the orthodontic tooth movement (OTM) process. Mechanical stress induces IL-1 beta release *via* EVs and IL-1 beta expression through Pannexin 1 and P2X7 receptor associated (Kanjanamekanant, et al., 2014).

##### 3.1.1.3 Dental pulp cells

Dental pulp cells (DPCs) are the predominant cells within the dental pulp of permanent teeth. DPC-EVs appear to be the potential biomimetic tool for tooth regeneration. First, EVs induce the odontogenic differentiation of stem cells, including pushing the differentiation of mesenchymal stem cells (MSCs) into odontoblasts and triggering the regeneration of dental pulp-like tissue *in vivo* (Couble, et al., 2000; Huang, et al., 2016). Exosomes bind linked to biomaterials even efficiently promote the formation of continuous reparative dentin in the minipig model of pulp injury (Wen, et al., 2021), which may be applied as a bioactive pulp-capping material in the future. Moreover, these vesicles also have vital roles in angiogenesis by promoting proangiogenic factor expression and tube formation (Zhou, et al., 2020), which are necessary for functional tooth regeneration.

##### 3.1.1.4 Odontoblasts and ameloblasts

Enamel and dentin are the primary hard tissue that make up the teeth. They are located outside of the dental pulp and play an essential role in protecting the entire tooth from external stimuli, especially the inflammation caused by caries (Kidd and Fejerskov, 2004). Previous findings suggest that these dental hard tissue-derived EVs may maintain tooth homeostasis by modulating the dentin crystal growth pattern and regulating enamel resorption and extracellular organic material digestion (Matsuo, et al., 1986; Rabie and Veis, 1995). Under healthy conditions, exosomes transport dentin phosphophoryn (DPP) to the extracellular matrix to construct well-mineralized tooth structures (Zhang, et al., 2014). Under diseases conditions, exosomes derived from severely inflamed odontoblasts attenuate apoptosis of mildly inflamed neighboring cells to protect the dentin (Wang, et al., 2019a).

##### 3.1.1.5 Stem cells from human exfoliated deciduous teeth

Deciduous and permanent tooth replacement is a special and actional process. It may take almost 6 years for humans to achieve the ordered transition of twenty deciduous teeth, which means that there are several opportunities to access sufficient Stem cell from exfoliated deciduous teeth (SHEDs) fairly easy in a period of up to 6 years. In addition to DPCs, SHEDs are another source of exosomes for dentine and dental pulp regeneration (Zhuang, et al., 2020). Moreover, SHED-EVs are even considered to provide an effective therapeutic tool in the treatment of Parkinson’s disease and TMJ inflammation, as their productive neuroprotective potential on human dopaminergic neurons and inflammation-suppressive potential on temporomandibular joint chondrocytes (Jarmalaviciute, et al., 2015; Luo, et al., 2019). The use of SHED-EVs, similar to the reuse of “biological wastes”, provides hope in zero biological cost regenerative medicine.

##### 3.1.1.6 Other cells

In addition to the above cells, several reports have shown that the oral cavity contains other uncommon cell sources of EVs, such as Hertwig’s epithelial root sheath cells (HERS) and human periapical cyst-mesenchymal stem cells (PCy-MSCs). HERS-secreted exosomes are a proven biomimetic tool in promoting odontogenic differentiation, neural differentiation, and tube formation *in vitro*, and regeneration of dental pulp-dentin-like tissue *in vivo*. Based on the roles of HERS in development, EVs might be considered as a mediator facilitating epithelial-mesenchymal interactions (Zhang, et al., 2020b). Researchers have found a novel MSCs community settled in the inner wall of dental periapical inflammatory cysts. Interestingly, naïve PCy-MSCs express primary neuronal markers and the main astrocyte markers. hPCy-MSC-derived EVs could also provide a wise “lab-on-cell” strategy to assess neurodegenerative disease therapies based on alterations in extracellular vesicle content (Tatullo, et al., 2019).

Furthermore, EVs have also been isolated from dental follicle cells (DFCs) (Shi, et al., 2020), oral keratinocytes (Sjoqvist, et al., 2019a), oral mucosa lamina propria-progenitor cells (OMLP-PCs) (Yang, et al., 2020a), and salivary gland epithelial cells (Kapsogeorgou, et al., 2005). However, EVs from these sources have not been broadly studied due to the particularity of the cells. Theoretically, EVs can be secreted by almost all types of cells. It is believed that a more extensive source of EVs will become available in the future.

#### 3.1.2 Bone marrow mesenchymal stem cells

Bone marrow mesenchymal stem cells (BMSCs) are highly effective cell sources of EVs. BMSCs can be harvested from maxilla or mandible bone marrow (Ding, et al., 2010), and can differentiate into osteogenic, chondrogenic, adipogenic, myogenic, or neurogenic lineages (Charbord, 2010). Based on the potent stemness of BMSCs, EVs have shown great potential for tissue regeneration and disease therapy. Several studies have illustrated that BMSC-EVs play crucial roles in vascularized bone regeneration (Liu, et al., 2021), cutaneous wound healing (Liu, et al., 2020b), cardiac functional recovery (Liu, et al., 2020c) and as immunomodulatory and anti-inflammatory agents for the management of periodontitis and colitis (Yang, et al., 2020b; Liu, et al., 2020d). These properties of BMSCs may provide advantages for EVs-based craniofacial tissue engineering and regeneration.

Bone is also a critical organ for the corresponding aging. *In vitro* experiments have shown that aged EVs are internalized by young bone marrow mesenchymal stem cells and inhibit the osteogenic differentiation of young BMSCs (Davis, et al., 2017). From this perspective, BMSC-EVs may have potential applications in antiaging interventions.

#### 3.1.3 Cancer cells

Similar to other cell types in physiological states, cancer cells in the oral cavity are also able to produce EVs in pathological conditions. To date, the majority of vesicles associated with cancer have been isolated from oral squamous cell carcinoma (OSCC) (Ono, et al., 2018; Zhao, et al., 2020) and adenoid cystic carcinoma (ACC) (Xu, et al., 2019; Chen, et al., 2020). It has been demonstrated that cancer cell-derived EVs can significantly increase cancer cell proliferation, migration, and invasion through the autocrine pathway, and the PI3K/Akt, MAPK/ERK, and JNK-1/2 pathways have been closely interrelated with EVs function in the tumor site (Sento, et al., 2016; Ono, et al., 2020). Simultaneously, as a critical component of the tumor microenvironment, EVs are highly involved in creating a favorable microenvironment to facilitate tumor progression and metastasis (Li and Nabet, 2019). It is worth mentioning that EVs also have “various tumor marker cargo” of early-stage tumor cells. Therefore, exosomes and other EVs are considered as potential biomarkers of liquid biopsy and may act as an innovative noninvasive diagnostic system for early cancer diagnosis (Zhang, et al., 2020c).

#### 3.1.4 Immune cells

Many chronic inflammatory diseases in the oral cavity such as periodontitis and oral mucosal infectious diseases often progressed by the acute immune responses to microbial. Previous investigations have demonstrated that a significant infiltration of immunes cells (*e.g.,* T cell and macrophage) in chronic inflammation of the gingiva, periapical tissues and alveolar bone (Hasiakos, et al., 2021). In inflammatory diseases, T cell-exosomes have been demonstrated to elicit the development of oral lichen planus (OLP) by increasing the infiltration of T lymphocytes in lesional sites (Yang, et al., 2020c). In the field of osteoimmunology, macrophage-derived exosomes have been shown to carry miR-378a to regulate the BMP2/Smad5 pathway for bone regeneration and even endogenous bone regeneration (Liu, et al., 2020e; Kang, et al., 2020). To pursue the optimal functionally engineered extracellular vesicles, researchers have designed T cell-derived chimeric apoptotic bodies (cABs) for on-demand inflammation regulation, which achieve accurate agent release at designated locations (Dou, et al., 2020). All these immune cell-EVs have shown diverse immunomodulatory properties and may act as modulators to affect the inflammatory response.

### 3.2 Extracellular vesicles released by cell-independent sources

#### 3.2.1 Microbiome

The dynamic microbiome in the oral cavity, mainly located in the saliva and plaque, is a direct cause of dental periodontitis and caries. Bacteria are the most common microorganisms in the oral cavity. It is worth noting that among extracellular vesicles, EVs derived from Gram-negative bacteria are often named outer membrane vesicles (OMVs) (Sartorio, et al., 2021). OMVs produced by *Porphyromonas gingivalis* (*P.g.*) (Sang, et al., 2014; Fleetwood, et al., 2017) and *Aggregatibacter actinomycetemcomitans* (*A.a.*) (Rompikuntal, et al., 2012; Nice, et al., 2018) are the most extensively investigated bacterial vesicles to date. Bacteria package and transfer genes and key virulence factors (LPS, gingipains, and fimbriae) through OMVs (Schwechheimer and Kuehn, 2015). Given the presence of the phospholipid bilayer membrane, these contents are protected from harsh conditions (Kadurugamuwa and Beveridge, 1999). Thus, bacterial EVs resemble a destructive bullet, promoting proinflammatory signaling cascades and long-distance microbiota-host communication (Jones, et al., 2020) to cause oral disease (Peng, et al., 2020) and systemic diseases such as Alzheimer’s disease (AD) (Singhrao and Olsen, 2018), neuroinflammatory diseases (Ha, et al., 2020b), infective endocarditis (Alkandari, et al., 2020) and respiratory system diseases (He, et al., 2020).

In addition to bacteria, the fungus *Candida albicans* is considered an important part of the healthy flora in the oral cavity. Once opportunistic infection occurs, it is often accompanied by oral candidiasis even potentially oral cancer (Lamont, et al., 2018). Although the mechanism(s) by which the fungal EVs across the thick cell wall remain unclear, *Candida albicans-*derived EVs have been identified and characterized, and were also shown to influence host immune response (Martinez-Lopez, et al., 2022), regulate biofilm formation (Honorato, et al., 2022), and confer drug resistance (Zarnowski, et al., 2018). State-of-the-art research suggests that EVs also play an important role in bacteria-fungi interactions and in turn impact oral disease progression. EVs derived from *Streptococcus mutans* augmented the virulence of *Candida albicans* by enhancing *Candida albicans* exopolysaccharides synthesis and biofilm development (Wu, et al., 2020), and subsequently increasing the dentin demineralization and *Candida albicans* cariogenic ability (Wu, et al., 2022a). These studies further suggest that extracellular vesicles released by the microbiome represent a novel potential target for the treatment of oral disease and system disorders.

#### 3.2.2 Saliva

Compared with the cellular supernatant, body fluids such as saliva, plasma, amniotic fluid, and breast milk are more common EVs sources. Ogawa, Y found exosome-like vesicles in human saliva for the first time and demonstrated that salivary EVs might play an administrative role in local immunity and participate in the catabolism of bioactive peptides (Ogawa, et al., 2008). Given the characterization of saliva, salivary vesicles show many advantages. Its collection is fast, simple, inexpensive, painless, and can be performed several times. In fact, the extracellular vesicles in saliva have diverse origins, including salivary glands, the oral flora, and any cells in the oral cavity. Furthermore, since EVs are believed to cross the epithelial barriers, which implies that they transport multitudinous components of systemic origin from the blood into saliva (Han, et al., 2018). Therefore, an analysis of EVs cargos circulating in salivary actually reflects the altered state of their diverse origins. Salivary EVs serve as a “mirror of the human body”, Proteins and miRNAs present in them offer insights into the clinical applications of oral diseases (Chaparro Padilla, et al., 2020), head and neck tumors (Yakob, et al., 2014), and systemic diseases (Kim, et al., 2017; Li, et al., 2020).

#### 3.2.3 Diet

The oral cavity is the start of the digestive tract, chewing and digesting numerous foods over an organism’s lifetime. Plant-based diets and milk are most frequently consumed foods. Researchers have found diet-derived miRNAs in mammalian cells and their modulation of mammalian genes, challenging the consensus (Yang, et al., 2015). A novel study has shown that milk-derived EVs have osteoprotective properties, these EVs initiate a decrease in osteoclast number, improving the bone microarchitecture (Oliveira, et al., 2020). Milk-EVs also reveal a potential capacity for involvement in embryo development (Sadri, et al., 2020) and immune regulation (Tong, et al., 2020). Edible plant-derived exosomes have been demonstrated as a potential therapeutic agent to treat periodontitis by significantly reducing the pathogenic mechanisms of *P.g*. (Sundaram, et al., 2019). These emerging findings may reveal a new potential source of EVs.

Extracellular vesicles have also been isolated from gingival crevicular fluid (Atsawasuwan, et al., 2018), junctional epithelium (Shimono, et al., 1991) and periosteum (Sun, et al., 2019). Perhaps because these sources are not representative, related studies are very limited. It is noteworthy that because of the sources and technical difficulty of exploring related research, cell-independent source is in its initial phases. Existing studies have shown that cell-independent sources mainly play a vital role in linking the oral cavity and systemic disease and may even be a mechanism of horizontal gene transfer. Thus, cell-independent sources are promising sources and cannot be ignored.

## 4 Biomedical applications of extracellular vesicles in the oral cavity

**FIGURE 3:**
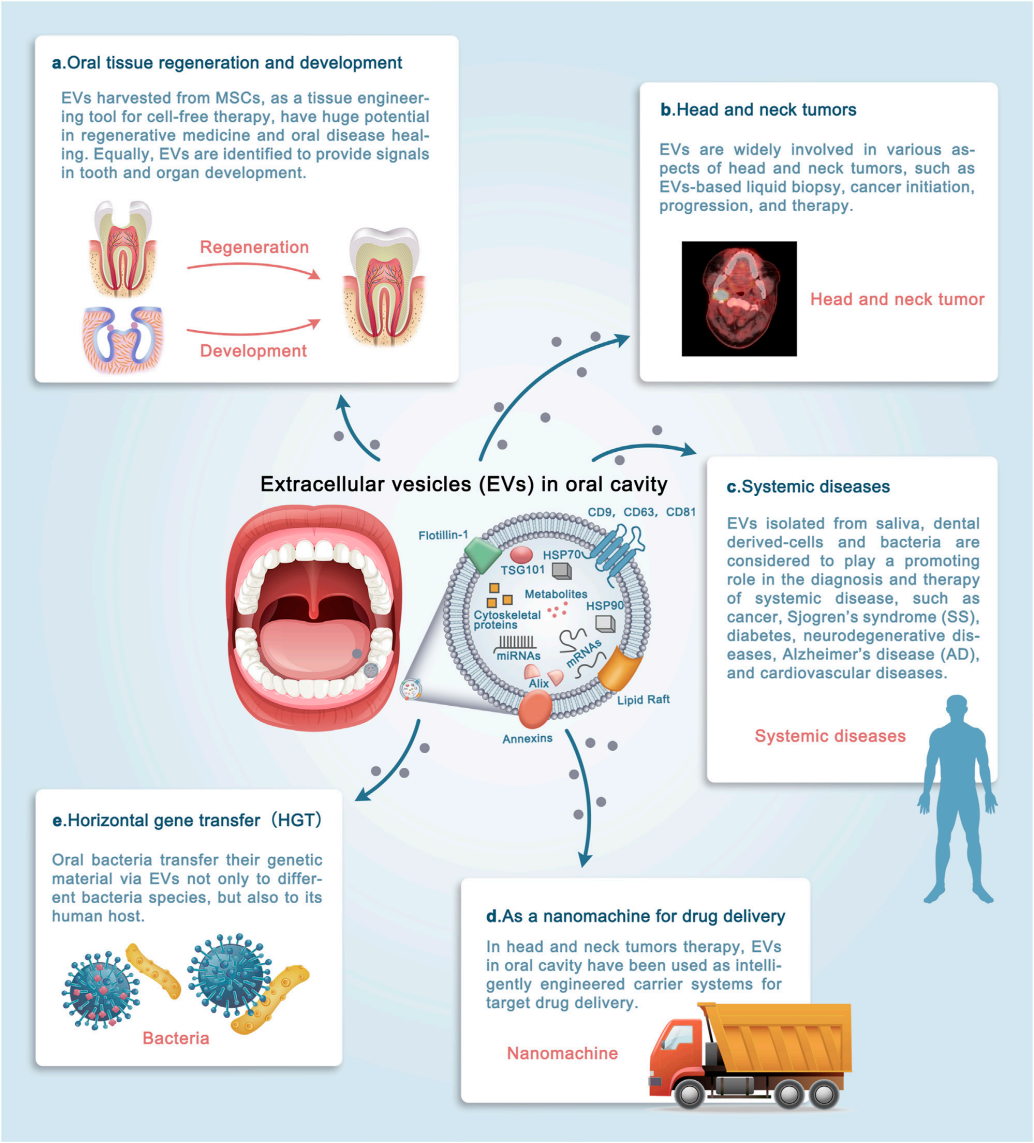
The roles of extracellular vesicles (EVs) in the oral cavity. The diverse sources of EVs in the oral cavity have allowed their involvement in a wide variety of physiological and pathological processes. **(A–E)** Schematic illustrating the promising clinical applications by which EVs may contribute to the diagnosis and treatment of diseases. Evidence supporting these applications is discussed in detail in the text. Note that EVs exert their function mainly in three ways: 1. source of EVs; 2. microenvironment in which EVs parent cells are located; 3. engineering of bilayer membrane vesicles structure. It is increasingly apparent that the clinical applications of EVs in the oral cavity are widespread, and the underlying signaling network needs to be deciphered. (MSCs: Mesenchymal stem cells).

Recent studies have shown that EVs exert their function mainly by transmitting their “cargo” to target cells, activating different signaling pathways, and modifying target cell biology (Raposo and Stoorvogel, 2013). The rich sources of EVs in oral cavity have made them acting different biological effects in many ways, and promising cell-free strategy for applications in clinical medicine. The function of extracellular vesicles in oral cavity will be reviewed in the following five sections from the perspective of clinical applications (Figure 3).

### 4.1 Application of extracellular vesicles in oral tissue regeneration and development

#### 4.1.1 Extracellular vesicles in craniofacial tissue regeneration

Extracellular vesicles harvested from dental tissue-derived cells have immense potential as a tissue engineering tool in regenerative medicine. Bone tissue engineering is the primary potential field. Exosomes have been demonstrated to accelerate bone formation even in rat osteoporosis, and this osteogenesis function will increase with the differentiation of osteoblasts (Wei, et al., 2019a). Mechanistic studies suggest that EVs may regulate angiogenesis-related genes (SDF-1, FGF2, and KDR), the BMP/Smad signaling pathway, and the Wnt/beta-catenin pathway (Wu, et al., 2019; Wang, et al., 2020b). The change of microenvironments in which parent cells are situated also impacts the osteoinductivity of EVs. Previous studies have shown that three-dimensional (3D) strain microenvironment influence PDLSC-derived exosomes, significantly improving the pro-osteogenicity of exosomes both *via* altering miRNA expression profile (miR-10a, miR-10b), and consequently improving the proliferation, migration, and osteogenic differentiation of target BMSCs (Yu, et al., 2021). The engineering of vesicles has further expanded their biomedical applications. EVs engineered with titanium nanotubes activate autophagy during osteogenic differentiation and show an effect on bone regeneration, thus facilitating the biofunctionality of titanium implants (Wei, et al., 2019b). Three-dimensional polyglycolic acid and polylactide (PLA)-engineered EVs (3D PLA-EVs) also show the activation effect of the osteogenic process (Diomede, et al., 2018).

Wound healing is another frequent medical problem. The facial skin and oral mucosa typically represent sites of injury, which may lead to an unattractive esthetic appearance and perhaps psychological issues. Several studies suggest that EVs are able to rapidly and substantially reduce wound size and structure (Sjoqvist, et al., 2019b). Apoptotic bodies derived from MSCs trigger the polarization of macrophages toward the M2 phenotype, and the resulting functional M2 phenotype increases the migration and proliferation of fibroblasts may represent the potential mechanism (Liu, et al., 2020a).

The tongue can be typically damaged in patients with oral cancer, but few studies have focused on tongue regeneration, especially tongue sensation. Surprisingly, EVs harvested from gingival mesenchymal stem cells are able to benefit taste bud regeneration and reinnervation (Zhang, et al., 2019). This impressive regenerative capacity may be related to the embryonic origin of oral tissue-derived cells is the neural crest (Stanko, et al., 2018). The similar potential of EVs in oral cavity for nerve be overlooked in neurological diseases.

#### 4.1.2 Extracellular vesicles in oral disease healing

Periodontal disease is a prevalent chronic inflammatory disease caused by microbe infection and destruction of tooth-supporting structures. Dynamic changes in the microbiome and phenotypic shifts of PDLSCs in the proinflammatory environment have led to the progression of periodontitis (Hasiakos, et al., 2021). EVs have been reported to have promising potential in periodontitis diagnosis and therapy. The detection of EV-related biomarkers in saliva will help to distinguish diseased from healthy tissues (Yu, et al., 2019). As periodontitis initiation and progression, the level of exosomes-based PD-L1 mRNA increased in saliva. Conversely, the level of miR-126 and miR-199a was reduced (Yu, et al., 2019; Nik Mohamed Kamal, et al., 2020). These findings highlight the potential of EVs as a biomarker in diagnosis. In view of microbes, vesicles are both a part and a toxic complex of bacteria. The vesicles derived from periodontitis-related bacterial species *P.g.* and *A. a.*, have been suggested to function as immunogens in developing periodontal disease vaccines (Sang, et al., 2014; Nice, et al., 2018). From the perspective of the inflammatory environment, vesicles secreted from MSCs and edible plants have shown strong modulation of inflammation and matrix degradation to prevent/treat periodontal disease (Sundaram, et al., 2019; Čebatariuniene et al., 2019).

**FIGURE 4:**
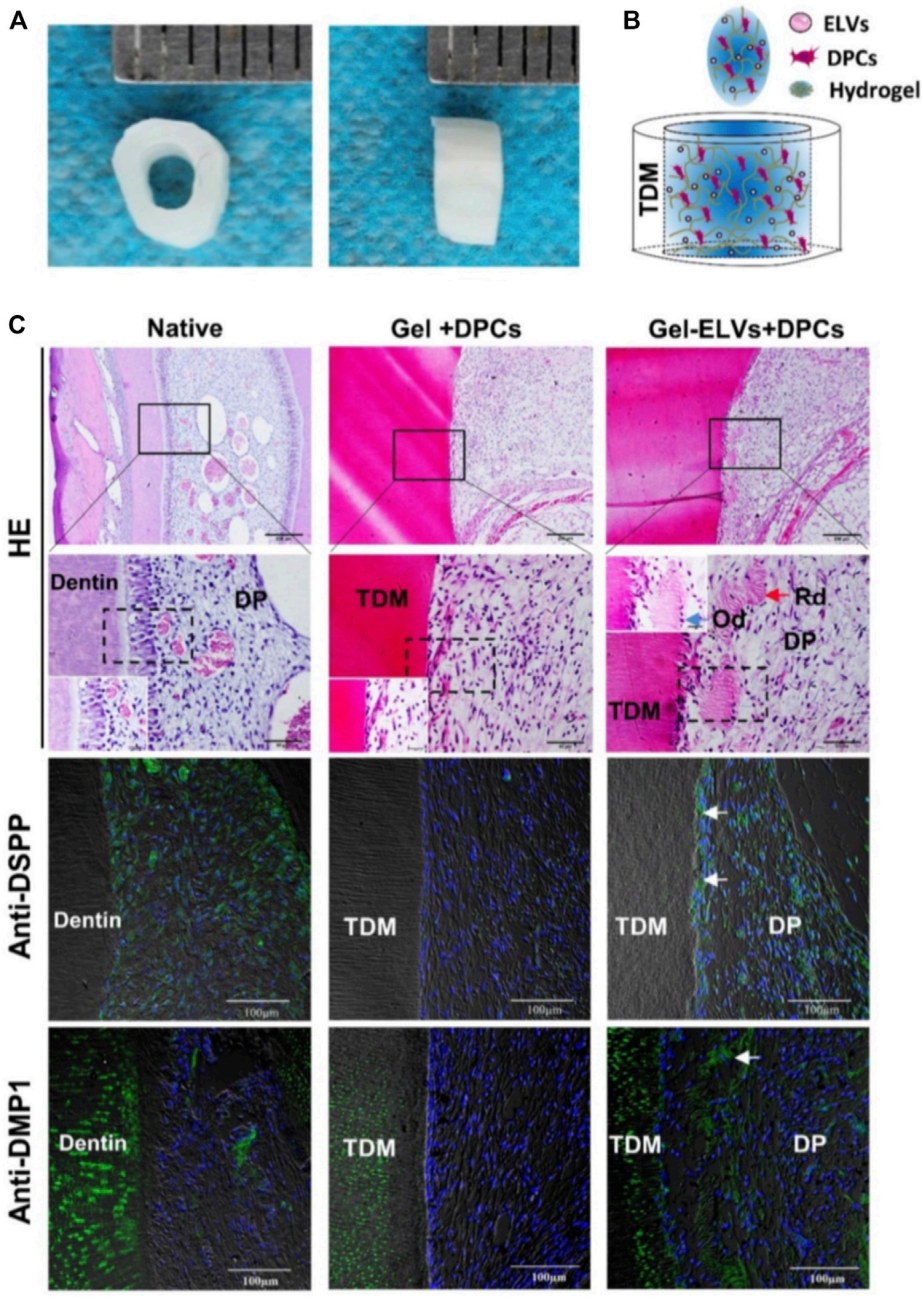
Hertwig’s epithelial root sheath cell-derived exosome-like Vesicles (ELVs) accelerate the formation of pulp-detin complexes *in vivo*. **(A)** The treated dentin matrix (TDM) canal obtained from extracted incisors of pigs formed a tooth root slice with an internal diameter of 2 mm and a height of 3 mm. **(B)** Schematic of the contents of TDM. Exosome-like vesicles were resuspended in DPC cells mixed with collagen hydrogel and then injected into the tooth root slice model. **(C)** HE staining showing newly regenerated tooth structures (e.g., polarizing odontoblast-like cells and predentin-like tissue) at the interface between the pulp-like tissue and the dentin. Immunofluorescence analysis revealing the increased expression of odontogenic differentiation markers (DSPP and DMP1) in the ELV-treated group, with white arrows indicating positive green staining. (DPCs: Dental pulp cells; DSPP: dentin sialophosphoprotein; DMP1: dentin matrix protein 1; Rd: regenerated dentin-like tissue; Od: odontoblast-like cell; DP: dental pulp-like tissue). Scale bars are shown. (Zhang, et al., 2020a Copyright; ivyspring).

Dental caries and endodontic disease are diseases that happen on the teeth themselves. Similar to periodontitis, bacteria are also an essential factor in the pathogenesis of dental caries. The short-size DNAs associated with *Streptococcus mutans* membrane vesicles, the principal pathogens of dental caries, are important contributors to the biofilm formation (Senpuku, et al., 2019). Thus, EVs may serve as an additional target for the prevention of caries. Irreversible pulp disease is another dismal disease. Once dental pulp is infected due to trauma or bacteria, the existing treatment such as root canal therapy cannot restore the function of the dental pulp and therefore causes a permanently devitalized tooth. Fortunately, exosome-like vesicles (ELVs) derived from Hertwig’s epithelial root sheath cells (HERS) significantly enhance pulp-dentin complex regeneration, as shown in the tooth root slice model in Figure 4 (Zhang, et al., 2020a). HE staining and immunofluorescence analysis demonstrated that HER-ELVs induced regenerated tooth structures (*e.g.,* polarizing odontoblast-like cells, predentin-like tissue, and collagen fibers) were close to the first molars levels of normal 4-week-old rats (Figure 4C). However, without ELVs, the Gel + DPCs group only displayed the formation of collagen and the deposition of extracellular matrix (ECM). The upregulated expression of dentin sialophosphoprotein (DSPP) and dentin matrix protein 1 (DMP1) indicated that ELVs treatment induced odontogenic differentiation in the tooth root slice model. Moreover, exosomes derived from DPCs and SCAP also show their potential on functional pulp-dentin complex regeneration.

Oral mucosal infectious diseases like leukoplakia and oral lichen planus (OLP) are potentially malignant oral disorders (OPMDs) that may develop into oral cancer. Investigators found that genetically modified EVs enriched with miR-185 are able to attenuate the malignant transformation risk of OPMDs by decreasing the inflammatory response and inhibiting cell proliferation and angiogenesis (Wang, et al., 2019b). Mucosal disease is often related to immune disorders. Circulating exosomes from OLP are not only able to be a biomarker for disease diagnosis (Byun, et al., 2015) but are also involved in the immunomodulatory functions of T cells (Peng, et al., 2019), suggesting a novel therapeutic option for oral mucosal disease.

The application of proper mechanical forces during orthodontic tooth movement (OTM) helps maintain periodontal tissue homeostasis and tissue remodeling surrounding the teeth. Recent studies have shown that EVs specifically express the miRNA-29 family during OTM in humans (Atsawasuwan, et al., 2018). Moreover, mechanical strain enhances the exosomal pro-proliferating effect *via* the miR-181b-5p/PTEN/AKT signaling pathway and enhances the osteogenic differentiation capability of PDLSCs by BMP2/Runx2, suggesting a potential mechanism for orthodontic tooth movement (Lv, et al., 2020). These studies on the character of EVs in OTM will afford novel insight that may identify means to achieving faster OTM procedures. Mechanical force (MF)-induced root resorption is a pathological side effect of orthodontic treatment, and the osteoclast and odontoclast biomarker RANKL in EVs may allow the detection of root resorption in the early stage to obtain a more secure orthodontic treatment (Cappariello, et al., 2018).

Bisphosphonates (BPs) are widely used in treating bone metastasis of cancer and osteoporosis. However, bisphosphonate-related osteonecrosis of the jaw (BRONJ) has been recognized as one of the most disabling disorders associated with BPs. Although little is known about the pathogenesis and treatment of BRONJ, Watanabe et al. showed that MSC-EVs are able to prevent BP-induced senescence and decrease the spread of chronic inflammation, thus promoting bone regeneration and preventing BRONJ (Watanabe, et al., 2020).

#### 4.1.3 Extracellular vesicles in tooth development

Recently, EVs were identified to provide tooth development signals based on the exquisite coordination of epithelial-mesenchymal interactions. Dentin matrix protein 1 (DMP1), present during the biogenic formation of mineral deposits, is a critical regulatory protein in dentinogenesis. It has been reported that EVs containing DMP1 are routed to the nucleus along microtubules during tooth development, and the GRP-78 receptor is also involved in this process (Ravindran, et al., 2008). Another dentinogenesis-related protein, dentin phosphophoryn (DPP), was demonstrated to be transported to the extracellular matrix through exosomes (Zhang, et al., 2014). For enamel development, Rab27a/b knockdown will alleviate EVs secretion and thus disrupt basement membranous formation and reduce enamel and dentin production (Jiang, et al., 2017). Additionally, vesicles are also observed in angiogenic regions, lining the luminal plasma membrane and formating capillary sprouts, which is a critical morphological event in dental organ development (Tsuzuki and Sasa, 1994). Mechanistically, the miRNAs in EVs influence development by regulating the expression of genes involved in DNA methylation in progenitor cells (Hayashi and Hoffman, 2017).

### 4.2 Application of extracellular vesicles in head and neck tumors

To date, application of EVs in oral cancer is the most investigated field of oral science. The tumor microenvironment is complex, with various cells and mechanisms involved. Previous reviews have detailed the role of extracellular vesicles in head and neck tumors (Xie, et al., 2019), so we just outline a few of important findings at this part.

EV-based liquid biopsy has unique advantages over traditional tissue biopsy as a noninvasive, real-time diagnostic technique (Colombo, et al., 2014). EVs provide better stability, contain high stocks of the original cellular biological information, thus providing better diagnostic accuracy. Substantial evidence has shown that salivary EVs are ideal diagnostic biomarkers. Compared with healthy populations, salivary exosomes show significantly increased concentrations, irregular morphologies, larger particle diameters and differentially expressed immune-related proteins in oral cancer patients (Zlotogorski-Hurvitz, et al., 2016). Recent studies have shown that miR-302b-3p and miR-517b-3p are expressed only in saliva EVs of oral squamous cell carcinoma (OSCC) patient. Excellent discrimination power for OSCC diagnosis based on exosomal miRNAs has been indicated by the ROC curve (Gai, et al., 2018). At the single vesicle and single protein levels, the significantly increased CD63 densities exhibited on the surface could also act as an index for cancer, even in the early stages (Sharma, et al., 2011).

Extracellular vesicles, which serve as intercellular information “trucks”, have great application potential in tumor therapy. To date, the application of EVs in clinical treatment can be divided into three areas. First, EV-based immune regulation methods. Programmed death-ligand 1 (PD-L1) is a crucial regulator by which tumor cells evade immunity. Compared with parent tumor cells, the level of PD-L1 in EVs significantly decreases in the early treatment stages and varies during anti-PD-1 therapy (Chen, et al., 2018). Second, EV-based drug-related methods. In view of the overall biocompatibility, membrane-based stability, and low immunogenicity of EVs, EVs can also be referred to as drug delivery vehicles. EVs have been used as a vehicle for the most common anticancer drugs of oral cavity cancer, such as doxorubicin (DOX) (Tian, et al., 2014), paclitaxel (PTX) (Batrakova and Kim, 2015) and curcumin (Sun, et al., 2010), increasing their therapeutic efficiency while reducing their side effects. Third, cancer cell-derived EVs are associated with decreased sensitivity of cancer cells to anticancer drugs. Downregulation of EVs may help solve the problem of anticancer drug resistance (Shedden, et al., 2003). It is believed that the application of EVs may be a promising field of cancer treatment due to their unique host fingerprint, ideal biocompatibility, and nanolevel molecular structure.

### 4.3 Application of extracellular vesicles in systemic disease

Accumulating evidence has revealed that extracellular vesicles derived from oral cavity are correlated with systemic diseases, such as Alzheimer’s disease (AD) (Hassan, et al., 2020), Sjogren’s syndrome (SS) (Kyriakidis, et al., 2014), foot-and-mouth disease virus (FMDV) (Wang, et al., 2020c), inflammation-related diseases (Pivoraite, et al., 2015), allergy-mediating diseases (Nazimek, et al., 2016) and aging (Machida, et al., 2015).

As an attractive EVs source with the advantage of noninvasiveness, human saliva is a unique medium for systemic disease diagnosis. Tumor-derived exosomes can be transported to and promptly detected in saliva upon cancer development. Like oral cancer, salivary EVs are a candidate for any cancer diagnosis (such as lung cancer, pancreatic cancer, and ovarian cancer) (Lau, et al., 2013). Sjogren’s syndrome is a common systemic autoimmune disease targeting salivary and lacrimal glands. The progressive damage of salivary glands will lead to dryness of the mouth and glazed tongue (Du, et al., 2019). Long-lasting, noninvasive, and more accurate diagnostic techniques are essential when evaluating primary Sjogren’s syndrome (pSS) patients. Research shows that salivary EVs can provide increased diagnostic accuracy in pSS (Li, et al., 2020) and can also be used to monitor the disease and staging (Aqrawi, et al., 2019). Altered bone metabolism as one of the long-term complications related to diabetes mellitus, usually increases alveolar bone loss and advances the progress of the periodontal disease. Due to the particular physiological condition of pregnancy, it often introduces difficulties in diagnosing and treating many diseases. Extracellular vesicles isolated from gingival crevicular fluid show a capacity to predict gestational diabetes mellitus in presymptomatic women (Monteiro, et al., 2019). Regarding technical aspects, the affinity chromatography column combined with a filter system (ACCF) as a simple approach has already been explored to efficiently remove the interference of saliva to obtain further purified salivary EVs (Sun, et al., 2016).

EVs isolated from dental tissue-derived cells and bacteria are considered to play a promoting role in systemic disease treatments due to the complexity of the oral environment and the special tissue origin of the oral cavity. Most dental MSCs exhibit convincing therapeutic functions in neurodegenerative diseases considering their neural crest cell origination. DPC- EVs even show the same neuroprotective efficacy as the neuron-MSC coculture system (Venugopal, et al., 2018). For specific diseases, PDLSC-EVs vesicles have been demonstrated to block experimental autoimmune encephalomyelitis and reverse disorder progression by reinforcing spinal cord integrity *via* remyelination (Rajan, et al., 2016). Among oral tissue, the dental pulp contains a rich nerve, this neuroprotective efficacy is also advantageous for the regeneration of functional tooth roots. The consensus that Alzheimer’s disease and periodontal disease have a bidirectional relationship means that treating periodontitis in AD patients will improve their memory. *Porphyromonas gingivalis* (*P.g.*) is the critical virulence factor of periodontitis. Recent studies indicated that the inhibition of *P.g.*, outer vesicles can modulate the progress of AD (Singhrao and Olsen, 2018). The membrane vesicles of *P.g.* also play a critical role in the pathogenesis of cardiovascular diseases by inducing inflammation (Yang, et al., 2016). These results suggest that the inhibition of EVs derived from bacteria will be a potential treatment target for several systemic diseases and systemic diseased-related oral diseases.

### 4.4 Application of extracellular vesicles as nanomachines for drug delivery

Given that EVs have relatively strong protective ability and ligand combining ability, it is hopeful that they will be developed into intelligently engineered nanovesicles for precision drug delivery. EV-based nanodrug delivery systems have multiple advantages over traditional drugs, including easier escape phagocytosis, enhanced targeting efficiency, prolonged drug release and reduced drug degradation (Vader, et al., 2016). Oral cancer-associated fibroblast-derived EVs have been used as a nanovehicle for miR-196a to mediate the cisplatin resistance of head and neck cancer, and this mediating effect is targeting CDKN1B and ING5 (Qin, et al., 2019). Additionally, encapsulation of anticancer agents such as paclitaxel into exosomes show significant accumulation in drug-resistant cancer and was 50 times more cytotoxic than conventional drugs *in vitro* (Kim, et al., 2016). It is worth mentioning that paclitaxel displayed the best potency to induce the apoptosis of oral squamous cell carcinoma cells compared to daunorubicin, doxorubicin and vincristine (Robert, et al., 2018). All the results imply the emerging strategy for EV-based therapy, may be promising for head and neck tumors treatment in the future.

Currently, oral drug delivery is the most commonly used administration system for the treatment of diverse diseases. Although EV-based targeted drug delivery systems overcome the limitations of traditional methods, they still have some barriers to clinical applications, such as difficulties harvesting sufficient EVs in a cost-effective manner. Recent studies have indicated that bovine milk may serve as a natural source for cost-effective and large-scale EVs production. Milk-derived EVs can be profitably used for oral drug administration of paclitaxel, affording desirable antitumor activity with high cross-species tolerance (Agrawal, et al., 2017). Further mechanism studies showed that the origin of distinguished oral-performance of milk-derived EVs including pH adaptation, intestinal mucus penetration and multi-targeting uptake (Wu, et al., 2022b).

### 4.5 Extracellular vesicles involved in horizontal gene transfer

Horizontal gene transfer (HGT) is the sharing of genetic material between organisms without a parent-offspring relationship. HGT is not only a widely recognized mechanism for drug resistance in bacteria but also builds the web of life even between multicellular eukaryotes (Soucy, et al., 2015). Several findings suggest oral biofilm-related bacteria release extracellular DNA (eDNA) *via* extracellular vesicles to other microbial (Liao, et al., 2014), and the EVs upregulate the transcriptional regulators expression of other microorganisms to influence the progression of caries (Wu, et al., 2020; Wu, et al., 2022a). Doxycycline resistance encoding transposons even can be transferred between different bacterial species *via* EVs in periodontitis patients (Warburton, et al., 2007). It is therefore plausible to speculate that EVs equipped with the specific antimicrobial target, may be promising for bacteria-associated caries and periodontitis treatment in the future.

The coevolution of the microbiome and its human host has led to refined interactions to maintain a sophisticated homeostasis. Studies on saliva indicate that *F. nucleatum* trigger the EV-mediated release of host transfer RNA-derived small RNAs (tsRNAs), and these tsRNAs in turn exerted a protein biosynthesis inhibition and growth inhibition effect on *F. nucleate* (He, et al., 2018). These findings suggest that the oral cavity as essential habitat for bacteria may also play a role in the long periods of evolution.

## 5 Conclusion and perspective

In this review, we focus on extracellular vesicles in the oral cavity. These EVs have several unique advantages, such as easy access, extensive sources, noninvasiveness, and broad application prospects. We discuss the extensive source of EVs in the oral cavity, including both cell and cell-independent sources. Then, the biomedical application roles of extracellular vesicles in craniofacial tissue regeneration and development, diagnosis and treatment of head and neck tumors, diagnosis and therapy of systemic disease, drug delivery, and horizontal gene transfer are introduced in detail. This paper represents the first effort on reviewing immune cells, odontoblasts, ameloblasts and diet EVs sources in the oral cavity, and applications of EVs in tooth development and bacteria mediated-horizontal gene transfer. Taken together, EVs in the oral cavity are a versatile communication device, and an in-depth realization of the source and roles of EVs under physiological/pathophysiological conditions may pave the way for the construction of diagnostic and therapeutic tools involving EVs. In the area of oral science, EVs still represent a most promising and exciting world that we are dedicated to pioneering.

However, several open questions remain to deserve further exploration. First, the oral cavity is an extremely complex environment that changes constantly. The influence and underlying mechanism of changing factors, including temperature, pH, oxygen, inflammation, and flora species, on EVs in oral cavity remain unclear. The second issue concerns the extracellular vesicles research itself. Supraphysiological numbers of cells were used to isolate EVs for experiments, but it remains unclear whether unpreconditional, physiological levels of EVs exert homeostatic or pathological functions (or neither) *in vivo*. Notably, the translation of findings from the laboratory to clinical practice is still challenging. The last but not least, our current knowledge of the cellular and molecular mechanisms that govern EV biogenesis, cargo sorting, release, and uptake remains limited. Inherent technical bottlenecks of precise isolating, quantifying, and characterizing pure populations of specific subtypes of EVs were a key limitation (Ramirez, et al., 2018). To facilitate and promote the EVs field, comprehensive studies in this EVs area are warranted. The safety, homogeneity, and standardized characterization of EVs should attract more attention in the future.
